# Case Report: First description of an intracranial “NET” in ROHHAD syndrome; time to rename to the ROHHAD-NeCT syndrome?

**DOI:** 10.3389/fendo.2026.1828990

**Published:** 2026-05-29

**Authors:** Nathalie J. Doelman-Oldenburger, Antoinette Y. N. Schouten-van Meeteren, Mariette E. G. Kranendonk, Kim Boshuisen, Michiel A. G. E. Bannier, Hanneke M. van Santen

**Affiliations:** 1 Princess Máxima Center for Pediatric Oncology, Utrecht, Netherlands; 2 Department of Pediatric Endocrinology, Wilhelmina Children’s Hospital, University Medical Center, Utrecht, Netherlands; 3 Department of Pediatric Pulmonology, MosaKids Children’s Hospital, Maastricht University Medical Center, Maastricht, Netherlands

**Keywords:** case report, hypothalamic dysfunction, intracranial tumor, neural crest tumor, neuroendocrine tumor, ROHHAD-NET

## Abstract

In around 50% of patients with Rapid-onset Obesity Hypothalamic dysfunction, Hypoventilation and Autonomic Dysregulation (ROHHAD) syndrome, a neuro-endocrine tumor (NET) is found. Reported NETS are (ganglio)neuroblastoma or ganglioneuroma located extracranially. Here, we report for the first time a young patient with signs and symptoms fitting the criteria of ROHHAD syndrome in whom an intracranial ganglion cell tumor was found, pathologically resembling the reported extracranial NETs. Our finding leads to the discussion of whether the NET in ROHHAD-NET syndrome should be classified as true NET or should be classified as Neural Crest Tumor (NeCT). Our case highlights the difficulty of the diagnosis of ROHHAD syndrome and the importance of screening for both extra- and intracranial Neural Crest Tumors in patients with rapid onset obesity at young age.

## Introduction

Rapid-onset Obesity with Hypothalamic dysfunction, Hypoventilation and Autonomic Dysregulation (ROHHAD) syndrome is a rare entity (1), of which currently only around 200 cases have been described in literature (2). It is important to recognize the disease, as it may be potentially life threatening due to cardio-respiratory failure. Diagnosis of ROHHAD syndrome, however, is hampered by the lack of a diagnostic test and is often a ‘per exclusionam’ diagnosis. To facilitate recognition and early diagnosis, clinical criteria to diagnose ROHHAD syndrome have been described (1, 3, 4). Children with ROHHAD are born healthy but develop rapid weight gain and obesity between the age of 1.5 and 7 years. The weight gain and subsequent obesity is caused by hypothalamic dysfunction, leading to hyperphagia and decreased energy expenditure (5). Typically, this rapid weight gain is followed by central hypoventilation, for which often ventilation during sleep is needed. In addition, other signs or symptoms of hypothalamic dysfunction may be present, such as pituitary dysfunction, and autonomic dysregulation leading to unstable body temperature and disrupted circadian rhythm (6). The onset of symptoms varies widely, which makes identification of patients with ROHHAD syndrome challenging. The pathophysiology of ROHHAD syndrome remains unknown (2, 7). In around 40-50% of patients with ROHHAD a specific type of neuro-endocrine tumor (NET), ganglioneuroblastoma, neuroblastoma or ganglioneuroma, is found, for which reason the syndrome is also known as ROHHAD-NET syndrome (1, 8). It has been hypothesized that these tumors give rise to an autoimmune or paraneoplastic phenomenon resulting in hypothalamic dysfunction (1). Ganglioneuroblastoma, neuroblastoma and ganglioneuroma derive from primitive neural crest cells that form the sympathetic nervous system. They are therefore found wherever sympathetic tissue naturally occurs. Usually, these tumors are located in the adrenal medulla, retroperitoneum or posterior mediastinum (9). Intracranial localization of a ganglioneuroblastoma, neuroblastoma or ganglioneuroma is very rare (10). We present the exceptional case of a patient with rapid weight gain, hypoventilation and an intracranial (non-hypothalamic) ganglion cell tumor in whom ROHHAD syndrome is suspected. To our knowledge, this is the first report of a ROHHAD patient with an intracranial ganglion cell tumor.

## Case description

A 22-month-old girl was admitted to the pediatric intensive care unit (PICU) due to respiratory failure and pneumonia. She was born at gestational age of 39 4/7 weeks, birth weight 2375 grams (<0.01^th^ percentile). She had a history of multiple clinical admissions due to RSV bronchiolitis, multiple wheezing episodes and unexplained obstructive apnea episodes. She developed obesity around the age of 1 year (BMI SDS + 2,32, with height SDS -0,28 and weight SDS + 1,65 (according to the Dutch growth charts (11, 12))), followed by further rapid weight gain before the age of 2 years (BMI SDS + 4,18, with height SDS -0,13 and weight SDS + 2,94). A CT-scan obtained as part of the workup for respiratory failure revealed mediastinal lymphadenopathy suspect for lymphoma. She was referred to the pediatric oncology clinic for further analyses and prednisone treatment was started (60mg/m^2^/day). In the PICU, labs were significant for a decreased free T4 and normal TSH (fT4 9.7 pmol/L (ref 12–22 pmol/L) with TSH 3.9 mU/L (ref 0.6-6.0 mU/L)). This was thought to be either concern for sick euthyroid or central hypothyroidism. The mediastinal abnormalities on the CT-scan decreased at repeat scans after four days and two weeks, lymphoma was excluded and steroid treatment was stopped after four days. Immunologic and infectious diagnostics showed no explanation for the lymphadenopathy or the presenting respiratory issues. She improved, however she continuously needed nightly supplemental oxygen.

Due to the combination of rapid onset obesity with hypoventilation and possibly central hypothyroidism a ROHHAD diagnosis was briefly considered. However, at this time not enough clinical signs were present. Abdominal ultrasound and CT-thorax showed no signs of possible neurogenic tumor related to ROHHAD.

Two months later, polysomnography not only revealed persistence of known obstructive apnea but also uncovered central apnea (Central Apnea Index (CAI): 36.1; Apnea Hypopnea Index (AHI): 66.5; Obstructive Apnea Index (OAI): 5.4). Targeted genetic diagnostics for genetic obesity, immune deficiency and developmental delay were negative. Additionally, exome wide copy number variant (CNV) analysis showed no genetic abnormalities, thereby excluding a PHOX2B mutation as well. Extensive pulmonary diagnostic work-up only showed signs of a post-infectious bronchiolitis obliterans for which she was treated with intravenous methylprednisolone pulse treatment. However, as no cause for the central apneas was found, a MRI brain was performed.

The MRI showed a large cerebellopontine mass suspect for a glioma (**Figure 1**). There were no abnormalities in the hypothalamic-pituitary region, the optic nerves nor signs of hydrocephalus on MRI. Ophthalmological evaluation showed normal eye motility and alignment; the optic nerves appeared normal although fundoscopy was difficult to perform. There were no new neurologic complaints. The tumor was not considered an explanation for her respiratory issues and also not for the development of obesity, for which reason the endocrinologist was consulted.

**Figure 1:**
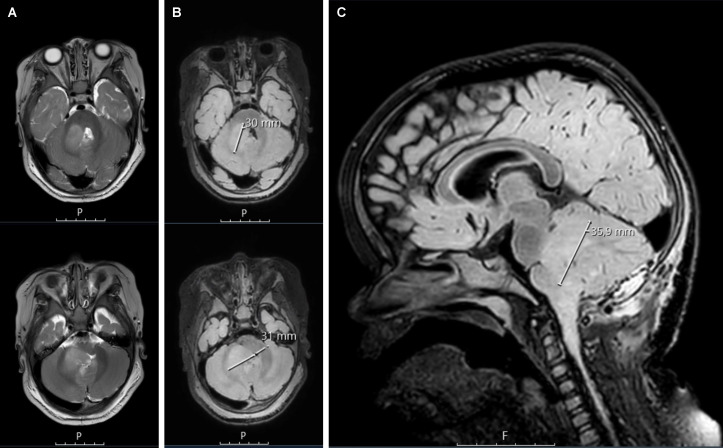
MRI images of the intracranial tumor. **(A)** T2 **(B)** MPR tra FLAIR **(C)** 3D FLAIR. Large hyperintense mass in right cerebellar peduncle, medulla oblongata, pons, cervical myelum. No hypothalamic or pituitary abnormalities are seen.

She underwent partial tumor resection with an uncomplicated post-operative course. Pathological investigation after neurosurgical partial resection showed a ganglion cell tumor; a low grade tumor with mostly ganglion cells expressing ganglion markers NeuN, neurofilament, chromogranin A and synaptophysin (**Figure 2**). In between the ganglion cells was a quite extensive lymphocytic infiltrate, and some stromal like cells focally positive for SOX10 (**Figure 2**). There was no clear glial component of the tumor (OLIG2 and GFAP-negative) and no neuroblasts were found. The tumor could not be classified using methylation profiling. With ddPCR a BRAF p.V600E mutation was found in 1.1% of the cells. While a BRAF mutation is typically found in ganglioglioma, a common CNS ganglion cell tumor, considering the histology and the clinical symptoms possibly fitting the diagnosis of ROHHAD-NET syndrome, this tumor was regarded as a neural crest tumor.

**Figure 2:**
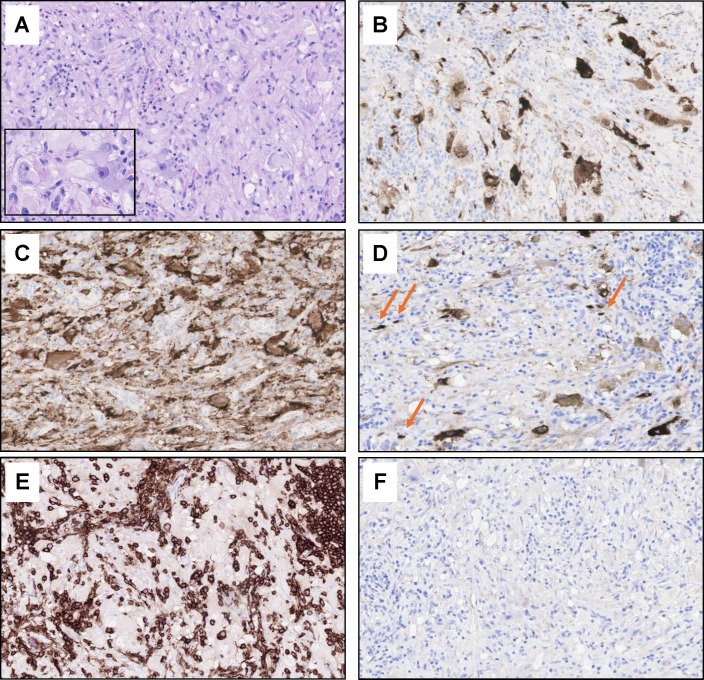
Histological and immunohistochemical characteristics of the tumor. **(A)** Hematoxylin and eosin staining, **(B,C)** immunoreactivity for chromogranin A and synaptophysin, respectively, **(D)** focal nuclear positivity for SOX10 indicating possible schwann-like cells (arrows), **(E)** CD45 indicating abundance of immune cells including ‘small blue round’ component, **(F)** negative for keratin. Magnification 100x, inlet 400x.

After tumor resection nightly respiratory support could be reduced from 24L/min to around 0,5L/min. Directly following the surgery the hyperphagia seemed to decrease, however this increased again during prolonged follow-up at 1,5 years post-surgery. Evaluation of the hypothalamic-pituitary function showed an elevated prolactin (0.84 IU/L, ref 0.1-0.65 IU/L) and reassuring and robust morning cortisol (0.48mcmol/L). There was no suspicion of growth hormone deficiency with IGF-1 concentrations reassuringly robust, although one time considered elevated at +2.1 SD. The higher IGF-1 may be related to the obesity, however during follow-up her IGF-1 concentrations normalized to between -1.60 and +1.60 SD. Her linear growth was normal within her target height range (height 98 cm, -1,0 SDS, target height SDS -1,1 at 1,5 years post-surgery). Thyroid function tests showed slightly elevated TSH values (11 and 13 mU/L, ref 0.35-5.0 mIU/L) with normal FT4 values (13–15 pmol/l (ref 12–22 pmol/l). Anti-TPO was negative. With persistent elevated TSH and low-normal FT4 suspect for either hypothalamic or mild primary hypothyroidism (13), thyroid hormone suppletion was started. Due to the combination of rapid-onset obesity at very young age, already before starting steroids, hypoventilation with central apnea’s, pituitary (hyperprolactinemia, possible central hypothyroidism) and hypothalamic dysfunction (temperature dysregulation, hyperphagia) (14), mild autonomic dysregulation (excessive sweating, ice-cold extremities with facial flushing) (2, 8) and now the finding of a ganglioneuroma type tumor, the diagnosis of ROHHAD-NET syndrome was considered likely.

Currently, she is three years after the initial neurosurgery. Obesity remains a major issue with current BMI at +4,8 SDS, with no steroid treatment, despite dietary intervention and strict follow-up. Her hypoventilation was initially stable, however recently her obstructive apneas have increased presumably due to the increase of obesity. An adenotonsillectomy was performed and nightly nasal CPAP started. A follow-up polysomnography hereafter showed an improvement of obstructive apneas, but persistence of central apneas. Currently, management with nightly CPAP seems insufficient and respiratory support with non-invasive ventilation may be started shortly. During the day, no respiratory support is needed. Mild hypothyroidism persists, which is treated with thyroid replacement therapy. The other pituitary axes are still intact. Other signs of hypothalamic dysfunction (5, 6) are consistently present, such as daytime sleepiness, temperature dysregulation with values of up to 35.5 and 39.6 degrees Celsius without signs of infection (ref 36.5-37.5), and minor behavioral issues. Her resting energy expenditure is recently shown to be decreased, which is typical for patients with hypothalamic dysfunction (15). Excessive sweating and ice-cold hands and feet with facial flushing remain present. When tired, she shows aggravation of her other symptoms of autonomic dysfunction such as aberrant eye position and torticollis. This is comparable to other patients known in our clinic with ROHHAD syndrome, although it must be considered that it might be related to the location of her tumor as well. The ganglion cell tumor is stable after the initial partial resection without any adjuvant therapy. In **Figure 3** her clinical timeline is summarized.

**Figure 3:**
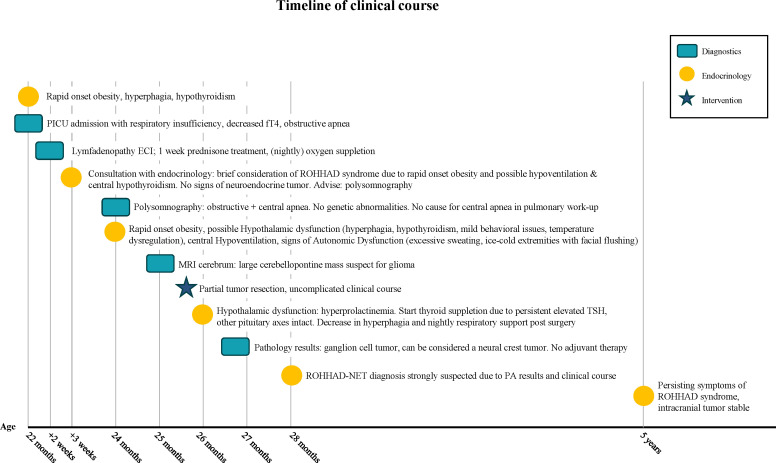
Summary of the clinical course of the patient.

## Discussion

This is the first report of a child suspected to have ROHHAD syndrome presenting with an intracranial neural crest tumor. ROHHAD syndrome is a rare disease and is difficult to recognize due to the lack of a diagnostic test. Usually children present with rapid weight gain or obesity, though they can also present with a ganglioneuroblastoma, neuroblastoma or ganglioneuroma. Signs and symptoms of hypothalamic or autonomic dysregulation may be mild initially (8). Early recognition of ROHHAD is essential as delay in diagnosis may increase the risk of cardiovascular complications and can even be lethal (16). For the acquired hypothalamic obesity promising pharmacological interventions are emerging, making timely diagnosis also relevant for the early treatment of rapid onset obesity (17). To aid in diagnostics, criteria for hypothalamic syndrome and hypothalamic dysfunction have been proposed in 2023 (6). Hypothalamic dysfunction may present with different clinical faces and therefore be difficult to recognize; pituitary function may even be normal (14). These suggested criteria for diagnosis of hypothalamic dysfunction in patients with tumors in the hypothalamic region may also be used in future patients presenting with rapid obesity at young age or with ganglioneuroblastoma, neuroblastoma or ganglioneuroma to improve recognition of the presence of hypothalamic dysfunction and, possibly, ROHHAD syndrome (6).

The diagnosis of ROHHAD syndrome is especially challenging since there is no cause yet identified for ROHHAD syndrome. A primary genetic basis has not been found, despite multiple studies investigating either specific candidate genes or including whole exome sequencing (2). The hypothalamic dysfunction with hypoventilation is thought to be caused by a paraneoplastic, autoimmune response causing hypothalamic inflammation. This is supported by the high incidence of neural crest tumors, similar symptoms occurring in opsoclonus myoclonus ataxia syndrome, and onset of disease in previously healthy children. Recently markers of inflammation and auto-antibodies such as ZSCAN1 antibodies have been identified in a few case reports, in patients both with and without a neural crest tumor, although these antibodies could not be detected in all individuals (18–20). In our case the combination of rapid-onset obesity at very young age, hypoventilation with central apnea’s, hypothalamic dysfunction (hyperprolactinemia, temperature dysregulation, hyperphagia) (14) and the finding of a ganglioneuroma type tumor, the diagnosis of ROHHAD-NET syndrome was considered likely. The hypothalamic dysfunction in our patient cannot be explained by an alternative diagnosis such as a suprasellar tumor, septo-optic dysplasia, or a genetic disorder like Prader-Wili syndrome. Congenital central hypoventilation syndrome has been ruled out by the absence of a PHOX2B mutation as a possible explanation for her central apneas. Our suspicion of ROHHAD syndrome has been confirmed during the last year with her decreased energy expenditure, persisting temperature dysregulation, progressive hypoventilation and symptoms of autonomic dysfunction such as aberrant eye position, torticollis and excessive sweating, comparable to other patients known in our clinic with ROHHAD syndrome.

The hypothalamic-pituitary dysfunction in patients with ROHHAD may vary from a (initially) normal pituitary function to panhypopituitarism with AVP deficiency and/or SIADH (4, 8, 14, 21). In our patient, we debated whether the thyroid function tests fit most with central hypothyroidism or with mild primary hypothyroidism. In a “non-hypothalamic suspect patient”, these lab tests would most probably fit with mild primary hypothyroidism. Patients with hypothalamic dysfunction may however also have moderate-elevated TSH concentrations (13). In our patient, thyroid function tests showed lowered FT4 values with normal TSH values possibly fitting the diagnosis of central hypothyroidism. In the course of time TSH values were mildly elevated with low-normal FT4 values for which thyroxine treatment was started. Due to the combination of rapid obesity with hyperphagia, hypoventilation and hyperprolactinemia and these thyroid function tests, hypothalamic hypothyroidism was thought to be plausible, although mild primary hypothyroidism cannot be excluded. However, the presence of pituitary-hypothalamic dysfunction in our patient is not debated, considering the occurrence of hyperprolactinemia as well as the other signs of hypothalamic dysfunction (6).

In the diagnostic work-up for presence of ROHHAD-NET, repeated screening for a NET is advised (2). In case of a child presenting with hypothalamic obesity, a brain MRI is recommended to exclude a suprasellar brain tumor or other damage to the hypothalamus as an alternative explanation for the clinical features (2). Our case is unique since a MRI was done to exclude brain stem abnormalities in the context of central apnea, and it was only then that we incidentally discovered her neural crest tumor in the cerebellopontine region.

The tumors reported in ROHHAD-NET comprise ganglioneuromas, ganglioneuroblastomas and neuroblastomas, which belong to the spectrum of peripheral neuroblastic tumors derived from neural crest cells. These tumors are characterized by varying proportions of differentiating neuroblasts, mature ganglion cells and Schwannian stroma. The presence or absence of a neuroblastic component distinguishes ganglioneuroma from (ganglio)neuroblastoma (22, 23). Importantly, these tumors do not correspond to neuroendocrine tumors (NETs) as defined by the WHO classification, nor the ENETS criteria (24, 25). According to these definitions, as used in pathology practice, the term NET is reserved for epithelial neuroendocrine neoplasms, defined by neuroendocrine morphology and expression of epithelial markers (CK8/CK18; CKAE1/3) and neuroendocrine markers such as chromogranin A and synaptophysin. While these tumors may produce bioactive peptide hormones or amines, hormone expression is variable and not required for diagnosis.

The intracranial tumor described here shows neuronal differentiation with expression of ganglion cell markers (including synaptophysin, NeuN and chromogranin A), however, in the absence of an epithelial component and is therefore not classifiable as a NET. CNS tumors with this morphology mostly resemble a ganglioglioma, especially in combination with the BRAF V600E mutation. However, this tumor does not show a glial component, therefore not fulfilling the essential criteria for ganglioglioma. Furthermore, there is a non-neuronal cellular/fibrotic component, focally showing nuclear positivity for SOX10, indicating possible Schwann-like cells. If this tumor were located in the adrenal gland, the tumor diagnosis would be ganglioneuroma, compatible with the ROHHAD tumor spectrum. However, its location in the CNS is not compatible with this tumor diagnosis and the tumor was therefore classified as a ganglion cell tumor.

Taken together, both the established literature and our findings highlight that the tumors associated with ROHHAD syndrome are more accurately conceptualized as neural crest–derived neoplasms rather than neuroendocrine tumors (2, 4, 8, 26, 27). We therefore propose that the acronym ROHHAD-NET be reconsidered and replaced by ROHHAD-NeCT (Neural Crest Tumor), which more precisely reflects the underlying tumor biology. The use of the term “NET” in ROHHAD-NET is potentially misleading, as it suggests a relationship to epithelial neuroendocrine neoplasia rather than to neural crest derived tumors. This distinction is further underscored by our case.

Therefore, this tumor is considered the first described intracranial neural crest tumor fitting ROHHAD-NET syndrome. A few cases of intracranial (ganglio)neuroblastomas have been described in literature, but no association with ROHHAD-NET syndrome has been made thus far (28–32). Two cases of intracranial ganglioneuroma at the pediatric age have been reported, with no description of ROHHAD-NET as well (10, 33).

In conclusion, this unique case illustrates how difficult it can be to make the diagnosis of ROHHAD syndrome. In the search for the neural crest tumor, it is important to be aware this may also be located intra-cranially. Additionally, the signs and symptoms of ROHHAD are easily missed by parents or caregivers if not evaluated explicitly. Combined with a current lack of awareness of the possibility of hypothalamic dysfunction in NeCTs this could lead to misrecognition of patients and delay in diagnosis, which could be detrimental. Further research is needed to improve recognition of hypothalamic dysfunction in patients presenting with rapid weight gain and/or with a neural crest tumor to identify the amount of patients with ROHHAD or ROHHAD-like phenotypes and improve timely diagnosis.

## Patient perspective

The period between admission to the PICU for respiratory insufficiency and the diagnosis of a brain tumor and ROHHAD syndrome was experienced by the family as a highly uncertain time. Although the patient initially showed clinical improvement, nightly respiratory support remained necessary. For the family, finding the underlying cause of these symptoms was the primary concern. The discovery of a brain tumor was entirely unexpected for them. However, quite soon the family considered themselves relatively ‘lucky’ regarding the tumor type, and realized that the tumor itself did not fully account for all the signs and symptoms the patient showed.

While ROHHAD syndrome is a serious and progressive condition, the family has expressed relief to finally have a diagnosis that could explain their daughter’s symptoms. They limit extensive reading about the syndrome, as published cases or information online usually describe more advanced disease stages or complications that the patient has not experienced thus far. Nonetheless, they see many of the described features in their daughter. Since the diagnosis, the family has connected with other (families of) patients with ROHHAD syndrome, finding peer support and recognizing many similarities in their experiences and phenotype. Finally, the family hopes that being diagnosed with ROHHAD syndrome may facilitate access to emerging and future treatment options for the patient. By sharing their daughter’s case, they wish to increase awareness of ROHHAD syndrome and of the possible association of ROHHAD with an intracranial tumor.

Written informed consent for the publication of this case report was provided by the patient’s mother.

## Data Availability

The original contributions presented in the study are included in the article/supplementary material. Further inquiries can be directed to the corresponding author.
